# On Syrup of Chloroform

**Published:** 1867-08

**Authors:** T. B. Groves


### *On Syrup of Chloroform.* By Mr. T. B. Groves, F. C. S.

* * * It was in attempting the preparation of chlorodyne, with a view of satisfying myself as to its reported difficulty of accomplishment, that I met with the facts forming the purport of this communication.

It has been proved by experiment that chloroform is soluble in water to the extent of two and a half minims per ounce, and that if a spirituous solution of chloroform containing a large proportion be added to water, the excess of chloroform soon finds its way to the bottom of the liquid, with which no amount of shaking will cause it to mingle sufficiently well to enable the dose to be accurately proportioned. * * * It occurred to me that if chloroform were reduced to exactly the same specific gravity as the syrup employed, by the addition of a liquid lighter than itself, mixture once effected would be permanent; there could apparently be no tendency to separation if the theory admitted of being practically carried out. It was obviously a *sine qua non* that the lighter liquid should not be liable to be abstracted by the syrup, or the chloroform would inevitably be precipitated in the globular form, as in the case of chloric ether.

I have succeeded in making such a mixture by reducing the specific gravity of the chloroform by means of ether, and shaking them with a definite amount of syrup. The chloroform manifests no tendency to separation, even when present in the proportion of one-eighth, but a better form is that containing one-twelfth.

The modus operandi is as follows:—Put into a twelve-ounce bottle one ounce of chloroform and about three drachms of ether; to the mixture add the same volume of the syrup to be employed; observe carefully the disposition of the fluids, the chloroform and ether will probably sink, then add *guttatim* more ether until the two liquids, on being shaken together, appear indifferent as to their position in the system; finally fill up the bottle with syrup, and shake well for a minute or two.

The syrup should not be too dense, or it will be difficult to impart to it sufficient agitation to ensure the complete commixture of the fluids. The syrup should be composed of gum and sugar, of honey or treacle; syrup of sugar does not answer well, apparently on account of lacking viscosity.

The syrup, thus formed, has the same physical properties as chlorodyne, and like it, is readily miscible with water in any reasonable proportion, (one to seven) and soluble in the water where the proportion of chloroform is within the limits of its solubility.

The advantages attending its use are these: 1. It does not need special precaution when being added to watery fluids, and in no case does it give rise to a deposition of large globules of chloroform. 2. When added in excess of saturation, the undissolved chloroform is deposited in *minute globules*, which, after lying together for days, show no disposition to combine, but may by a few shakes be dispersed evenly through the liquid, forming an emulsion sufficiently permanent to enable a dose to be measured without difficulty. (*London Pharm. Journal*, *June*, 1864). (*Detroit Review of Medicine and Pharmacy*). *Journal of Materia Medica.*

